# Sample cell for studying liquid interfaces with an *in situ* electric field using X-ray reflectivity and application to clay particles at oil–oil interfaces

**DOI:** 10.1107/S1600577518004848

**Published:** 2018-04-24

**Authors:** Simon R. Larsen, Marie Hansteen, Barbara Pacakova, Keld Theodor, Thomas Arnold, Adrian R. Rennie, Geir Helgesen, Kenneth D. Knudsen, Heloisa N. Bordallo, Jon Otto Fossum, Leide P. Cavalcanti

**Affiliations:** a Niels Bohr Institute, University of Copenhagen, Universitetsparken 5, Copenhagen 2100, Denmark; b Norwegian University of Science and Technology (NTNU), Trondheim 7491, Norway; c Diamond Light Source, Harwell Science and Innovation Campus, Fermi Avenue, Didcot OX11 0DE, UK; d European Spallation Source ERIC, PO Box 176, SE-221 00 Lund, Sweden; e Centre for Neutron Scattering, Uppsala University, Box 516, Lägerhyddsvägen 1, Uppsala 75120, Sweden; f Institute for Energy Technology (IFE), Instituttveien 18, Kjeller 2007, Norway

**Keywords:** X ray reflectivity, electrical field, liquid surface, oil–oil interface, clay

## Abstract

Commissioning results of a liquid sample cell for X-ray reflectivity studies with an *in situ* applied electrical field are presented. The cell consists of a Plexiglas container with lateral Kapton windows for air–liquid and liquid–liquid interface studies, and was constructed with grooves to accept plate electrodes on the walls parallel to the direction of the beam. Both copper and ITO plate electrodes have been used, the latter being useful for simultaneous optical studies. Commissioning tests were made at the I07 beamline of the Diamond Light Source.

## Abstract 2

A sample cell for studying liquid interfaces with an *in situ* electric field using X-ray reflectivity and its application to clay particles at oil–oil interfaces is described.

The adsorption of colloidal particles at the surface of liquid droplets is used in particle-stabilized surfactant-free or Pickering emulsions (Schmitt *et al.*, 2014 ▸; Gonzalez Ortiz *et al.*, 2017 ▸), with wide applications in pharmaceutics and the oil industry. Clay particles driven by an electro-hydro­dynamic convective flow were used to coat a silicone oil drop immersed in castor oil (Dommersnes *et al.*, 2013 ▸), which was dynamically modulated by tuning the external electric field strength. The interface deformation that causes the ordering of the particles is related to the redistribution of charges of the colloidal particles induced by a non-polar surface, like oil or air (Nikolaides *et al.*, 2002 ▸). Additionally, it is known that colloidal particles can be trapped in a surface energy well at the water–air interface and organized in a two-dimensional lattice (Pieranski, 1980 ▸).

To contribute to the understanding of the stability of particles at liquid interfaces under applied electric fields, we have constructed a sample cell to study the ordering of clay confined in two dimensions at the interface of two oils using synchrotron X-ray reflectivity (XRR) with an *in situ* applied electrical field. Our sample cell for liquid–liquid interface studies, built at the University of Copenhagen, was made of Plexiglas using lateral Kapton windows for the incoming beam to reach the liquid–liquid interface, and with grooves on the walls parallel to the direction of the beam for placing electrode plates for application of an electric field only along the *x* direction, as shown in Fig. 1(*a*) ▸. Two sets of electrodes, copper and indium tin oxide (ITO), can be used, the latter for simultaneous optical studies.

The commissioning of the sample cell was performed at the Diamond beamline I07 designed for surface and interface diffraction (Nicklin *et al.*, 2016 ▸) and equipped with a double crystal for deflecting the incoming beam onto a liquid surface (DCD mode) (Arnold *et al.*, 2012 ▸). We used a Pilatus-100k detector with sample-to-detector distance of 0.8 m and beam energy equal to 24 keV in order to achieve a good transmission through the oil media as estimated using an optical path of 110 mm of oil and the measured flux of I07 (Nicklin *et al.*, 2016 ▸) as shown in Fig. 1(*b*) ▸.

The length of the sample cell, 110 mm along the incident plane, was chosen in order to use the whole footprint of the incoming beam (vertical size ∼60 µm) at an angle of incidence of ∼0.1°, considering just the flat part of the sample, without the meniscus region. The sample consisted of a suspension of 0.125% (*w*/*w*) lithium fluoro­hectorite (Li-Fht) clay particles in silicone oil deposited on castor oil. As the system had a meniscus on the Kapton window that curves up, the incoming beam was incident from underneath the interface created by the precipitation of the clay particles, as shown in Fig. 2(*a*) ▸.

A snapshot of the measured reflected beam at the detector and the corresponding reflectivity curve from the oil–air interface are shown in Figs. 2(*b*) and 2(*c*) ▸, respectively. A similar response would be expected for an oil–oil interface in this geometry. The scattering length densities of the chosen oils are very similar and so we expect to have an enhanced signal from the clay particles at the oil–oil interface. Data analysis is ongoing.

The ordering of the clay particles at the interface, shown in Fig. 3 ▸, will depend on a delicate balance between electric and capillary forces. By applying an electric field, the particles will be polarized, and thus arranged into different patterns such as clusters (Fig. 3*a* ▸) or aligned bead chains (Fig. 3*b* ▸). Studies of this are ongoing. The cell was tested in the 0–3 kV range (nominal) across a gap of 15 mm in the *x* direction, which gives a maximum static field of 200 V mm^−1^, homogeneous in the area illuminated by the X rays.

This new cell is suitable for air–liquid and liquid–liquid interfaces and opens the opportunity for XRR studies on interface systems requiring *in situ* electric fields, and in particular for studying particles for electrically controlled self-assembled surface structures.

## Figures and Tables

**Figure 1:**
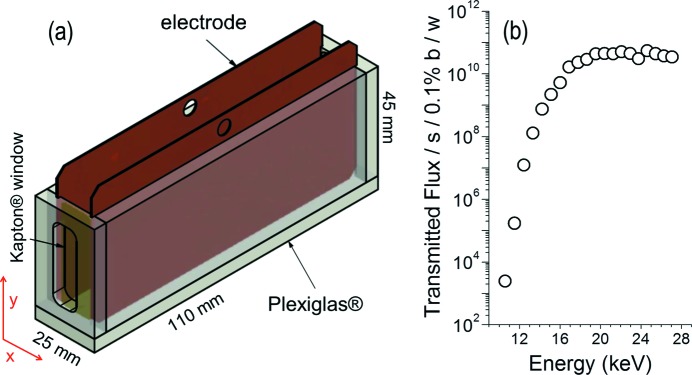
(*a*) Drawing of the sample cell for air–liquid or liquid–liquid XRR experiments. (*b*) Transmitted flux of beamline I07 estimated for a path length of 110 mm of castor oil inside the sample cell.

**Figure 2:**
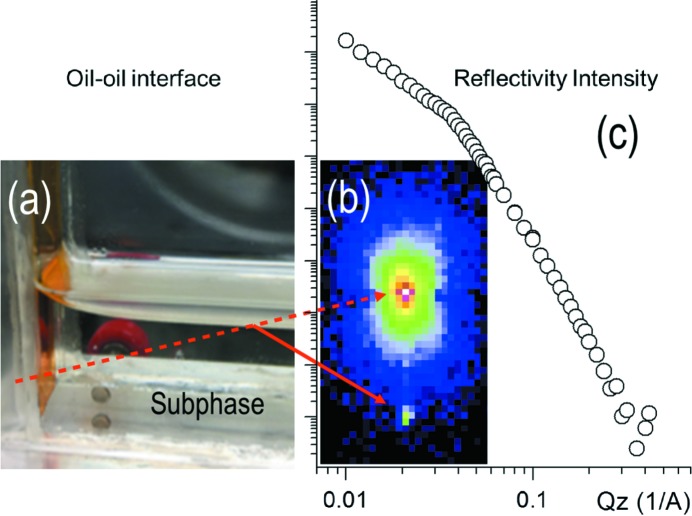
(*a*) Photograph of the sample cell showing the interface between castor oil (subphase) and the Li-Fht clay suspension in silicone oil. The red dashed and continuous lines represent the direct and reflected beam, respectively. (*b*) Detector image showing one snapshot of the direct (refracted) beam and the reflected beam. (*c*) Reflectivity recorded from the oil–air interface, which is not expected to have a critical edge and the intensity axis has no absolute normalization. The inflection point seen in the curve is likely to be due to contamination of the measured reflectivity from the direct beam as the angle is reduced and the two spots on the detector converge (data reduced using the *RODAN* package with *DAWN* at Diamond).

**Figure 3:**
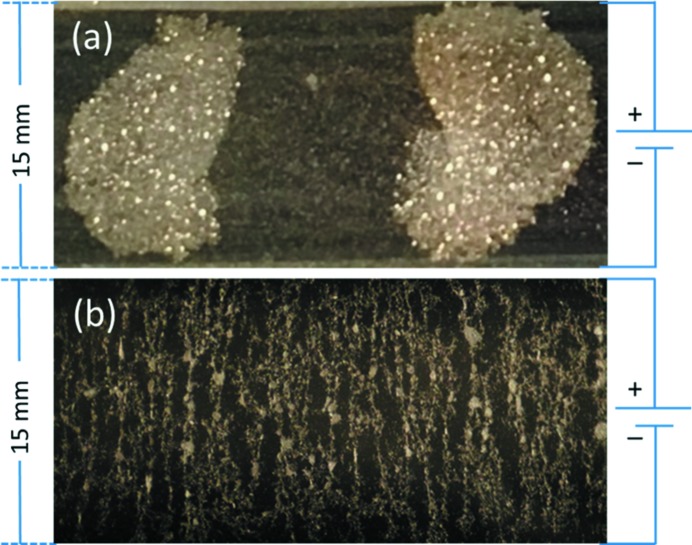
Top view of the sample cell showing the Li-Fht clay particles assembling at the oil interface under an *in situ* applied DC electric field of 200 V mm^−1^. Both (*a*) clusters and (*b*) bead chains assemble in alignment with the electric field.
